# Supplementary material for: Coronavirus-like all-angle all-polarization broadband scatterer
Source: Commun Eng. 2023 Oct 24;2:76. doi: 10.1038/s44172-023-00116-w (PMC10955951; doi:10.1038/s44172-023-00116-w)
Supplement: Supplementary file 2 — Supplementary Information [file 44172_2023_116_MOESM2_ESM.pdf]

Coronavirus-like All-angle All-Polarization Broadband Scatterer Supplementary Information

Anna Mikhailovskaya[1,a), =], Dmytro Vovchuk[1, =], Konstantin Grotov[2], Denis S. Kolchanov[1], Dmitry Dobrykh[1], Konstantin Ladutenko[2], Vjačeslavs Bobrovs[3], Alexander Powell[4], Pavel Belov[2] and Pavel Ginzburg[1]

[1]School of Electrical Engineering, Tel Aviv University, Tel Aviv 69978, Israel

[2]School of Physics and Engineering, ITMO University, St. Petersburg 197101, Russia

[3] Institute of Telecommunications, Riga Technical University, Riga, Latvia

[4]Department of Physics and Astronomy, University of Exeter, Exeter EX4 4QL, United Kingdom

Supplementary Note S1. Scattering cross-section dependence on the angle of incidence and polarization

To justify the all-angle-all polarization response, we made a set of numerical simulations. The results are summarized in Fig. S1. The inset demonstrates the arrangement – the wave is incident from either X, Y, or Z direction and has different polarization states, which are indicated in the legends. For the sake of clarity, the polarization states are – $(E_o\cos(\alpha)\,\hat{x} + E_o\sin(\alpha)\hat{y})\cdot\exp(ik_oz)), \alpha = 0°, 15°, 30°, 45°, (E_o\hat{y}\cdot\exp(ik_ox)),\ (E_o\hat{z}\cdot\exp(ik_oy))$ – from the top to the bottom in leggings. It can be seen that the variations over the spectrum do not exceed 2-3%. The same procedure was performed in the experiment and no significant variations (above signal-to-noise) have been noticed.

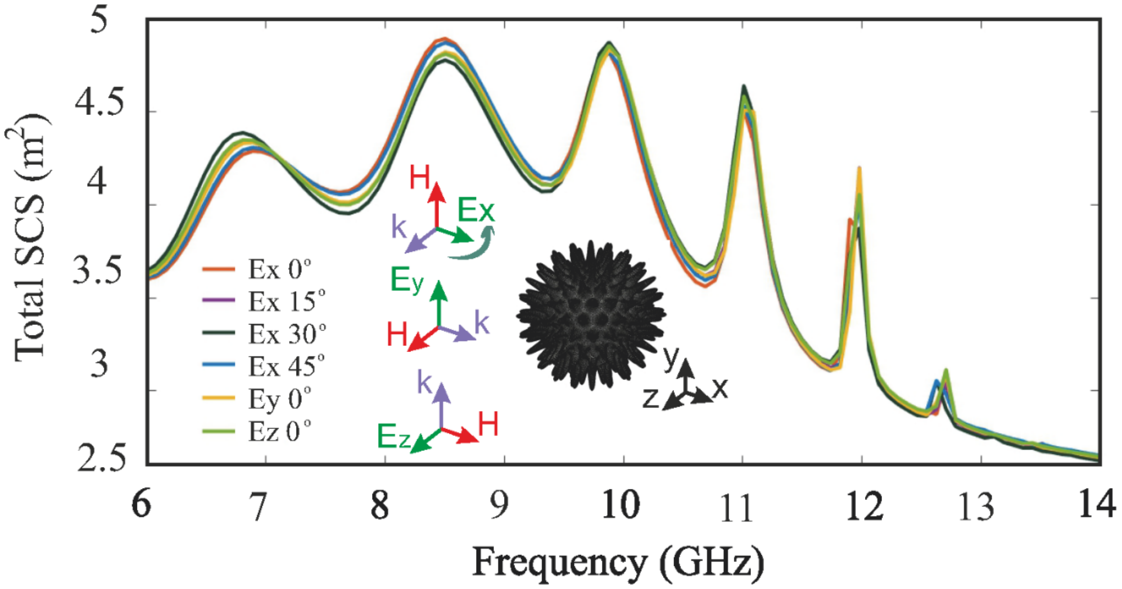

Figure S1. All-angle all-polarization analysis – scattering cross-section spectrum for different excitations (in legends).

Supplementary Note S2. Corona Scatterer construction

Step 1. Creating the Icosphere	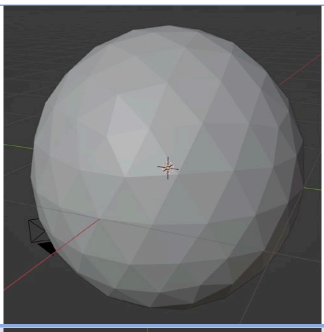
Step 2. Hexagons or pentagons are created in vertexes, depending on the number of edges) with specified area	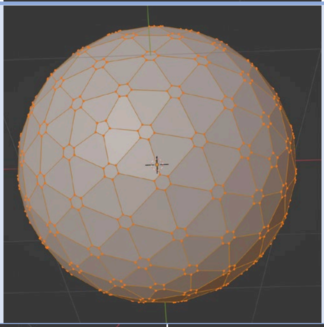
Step 3. Polyhedrons extrusion to a specified height.	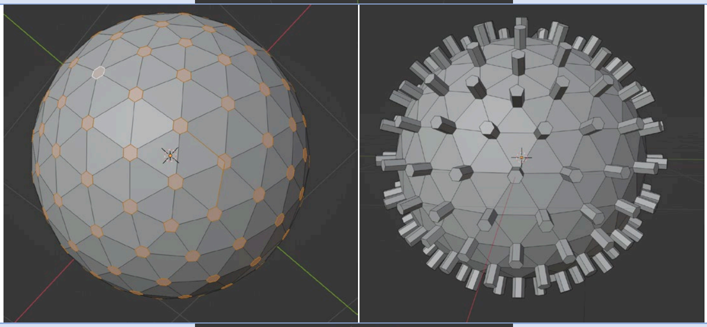
Step 4. Creating a difference of widths on the bottom and top of the spikes	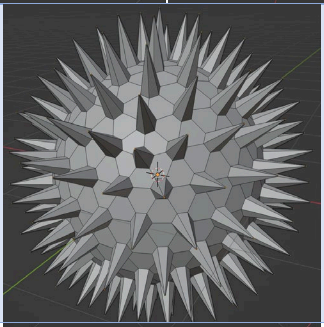
Step 5: Smoothing the resulting corona scatterer using Catmull–Clark subdivision surface algorithm	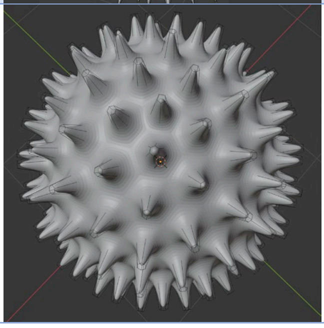

Supplementary Table 1. The main steps of the scatter's construction.

Blender script https://doi.org/10.5281/zenodo.6962448

Dataset is available online on Zenodo: https://zenodo.org/record/6962448#.ZA8jvS8w2Lc

Supplementary Note S3. Investigation of fabricated samples surfaces

Surface roughness and imperfections on scales, which are much smaller than operational wavelengths, weakly affect the electromagnetic performances of metalized layers (e.g., [1,2]). Fig. S2 demonstrates microscope images of the most damaged parts, found on the samples. An additional assessment was made on DC conductivity using a KEITHLEY 2700 multimeter. In this case, multimeter probes were attached to the opposite poles of the samples, resistivity was recalculated in (Ohm × cm) in accordance with the geometry of the sample.

Samples obtained by the electroless deposition method (Fig. S2 (a)) show the lowest resistivity of about 15 ± 3 Ohm × cm. In addition, the adhesion to the sample surface is insufficient to pass the adhesive tape test. In this case, the conductivity of the sample strongly depends on scratches and inhomogeneities. The main source of scratches is associated with the possible deformation of the PLA plastic during sample preparation and its subsequent drying. The bumps and inhomogeneities are the results of a nonuniform or insufficient number of nucleation centers (Pd^{2+}). Further efforts on surface treatment can improve the adhesion of the copper layer. Regardless of the numerous technical aspects, this method is very attractive in case massive manufacturing of samples is required.

Samples with a magnetron deposition of gold (Fig. S2(b)) demonstrated a moderate resistivity of 0.9 ± 0.3 Ohm × cm. Owing to the complex topology of the sample, there are small islands that are not perfectly covered with gold. In addition, cracks could appear during the deposition process due to the immersion of plastic in the high vacuum, which is required for the operation of the magnetron sputter. Due to the presence of the chrome adhesive layer, gold shows high adhesion to the sample. As a result, all samples obtained by this method easily pass the adhesive tape test. Based on the obtained results, this method provides highly sustainable samples, though the production cost can be quite high.

Electrical deposition of copper (Fig. S2 (c)) shows the best result in resistivity. According to the measurement, it is less than 100 μOhm × cm (our measurement apparatus has no sensitivity to go below this value). The sample shows excellent adhesion according to the tape test and seems to be the most promising. This approach is the unambiguous compromise for the reported application.

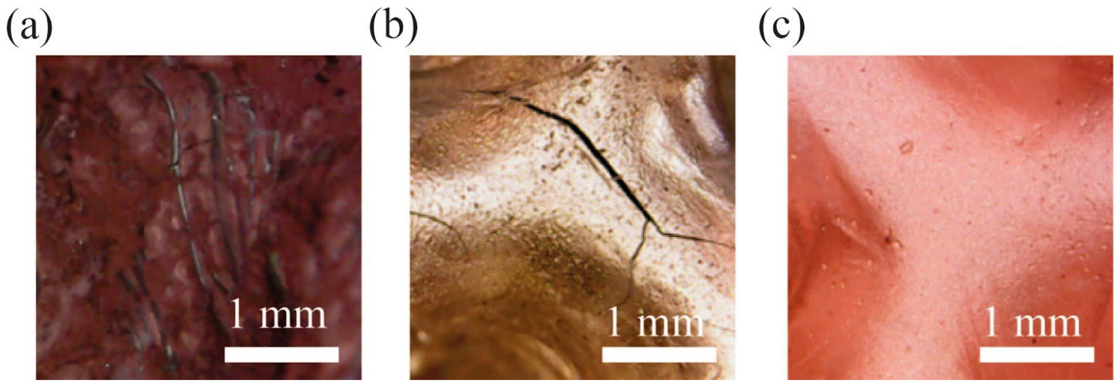

Figure S2. Zoom on samples' surface cracks. (a) Electroless deposition of copper. (b) Magnetron sputtering of gold. (c) Electrodeposition of copper

Supplementary Note S4. Creeping waves on different geometries

Creeping waves are not eigenmodes of the structure, nevertheless, they are used as a descriptive tool to explain returns from radar targets. In smooth geometries, the resonant reflection is typically related to the circumference of the scatterer. In the case of complex topology, the group delay can appear owing to structuring. Supplementary Table 2 provides the results on basic structures, which were considered to summarize the methodology. The top panel demonstrates the pulse reflection from a flat surface. No significant spitting was observed. The middle structure is the same 100x100mm thin copper sheet but illuminated from the side. The field is polarized perpendicular to the plane. The reflected pulse splits into – the specular reflection and the creeping wave. The delay between those two corresponds to the physical distance traveled along the circumference of the structure. The last panel demonstrates the scenario, where the wave interacts with a smooth copper sphere ($2\pi r$ = 125 mm). Copper electrodeposition on 3D-printed PLA sphere was used as a fabrication method.

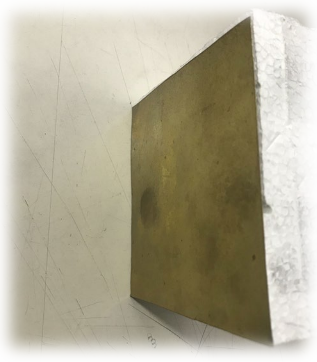

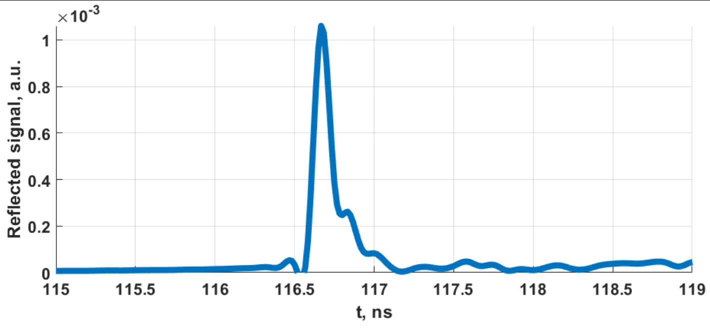

Normal incidence, no creeping wave

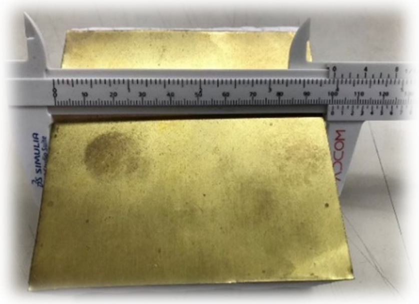	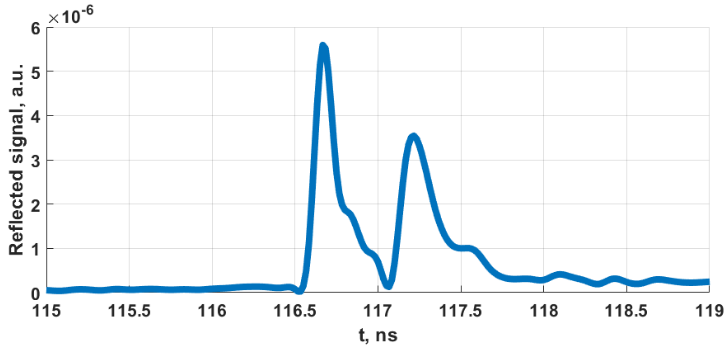<br>$\tau \approx 0.6ns;\ \ d = c\tau \approx 180mm$
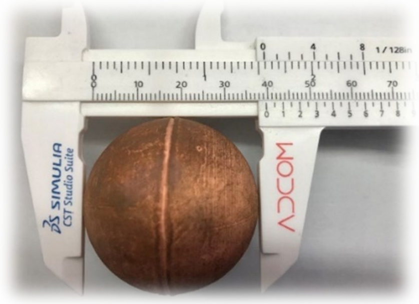	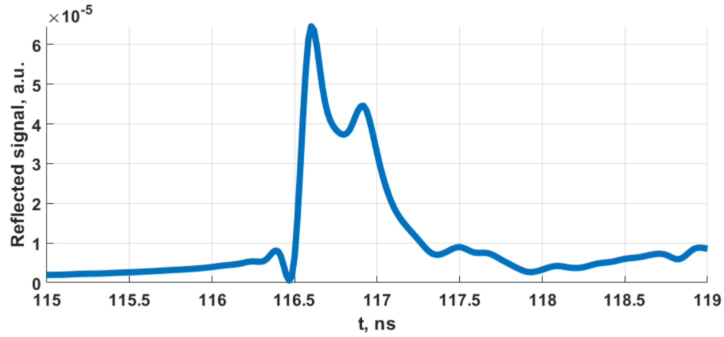<br>$\tau \approx 0.33ns;\ \ d = c\tau \approx 100mm;\ \ d = \pi R + 2R$

Supplementary Table 2. Experimental demonstration of creeping waves. Structures – left column, reflected pulse - right column.

Supplementary Note S5. Impact of Spike height in the scattering cross-section

To verify the contribution of spikes on the scattering cross-section, we performed a parametric study, which results are summarized in Fig. S3. It can be seen that for relatively short spikes the scattering cross-section approaches this of a smooth metal sphere. Increasing the lengths of spikes changes the performance. Intermediate values (0.3 – refer to the geometrical definitions in the main text) primarily affect the high-frequency region, while long spikes contribute to the lower frequencies. As a result, the bandwidth can be optimized with this parameter. It is worth noting that for a fair comparison, the minimal radius of a virtual sphere, encompassing the structures, is kept the same.

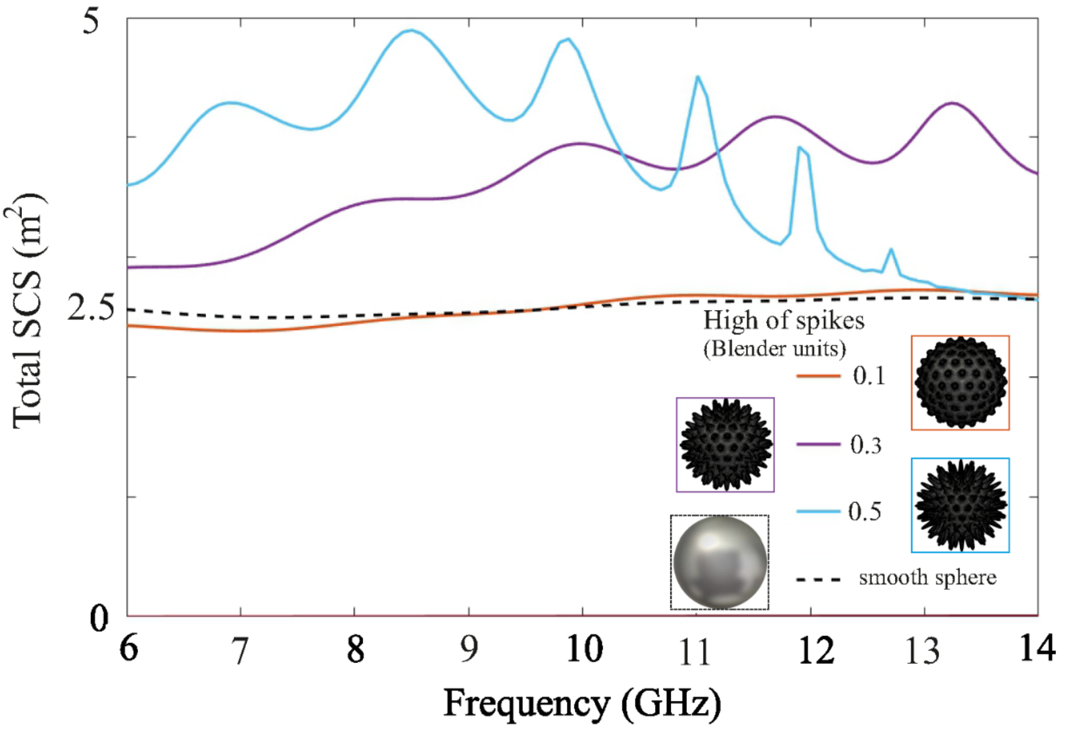

Figure S3. Scattering cross-section for structures with different heights of spikes (in legends).

Supplementary Note S6. Multipole Expansion

To reveal the device operation, multipolar expansion of the scattering spectra should be done. In this case, the finite element method (COMSOL Multiphysics) was used. The multipole expansion of the scattering cross-section, i.e., the sum of the contributions from different multipole moments (Cartesian multipoles were used here) up to the third order is given by [3–6]

$$\sigma_{sca}^{tot} = \sigma_{sca}^{p} + \sigma_{sca}^{m} + \sigma_{sca}^{Q^e} + \sigma_{sca}^{Q^m} + \sigma_{sca}^{O^e} + \sigma_{sca}^{O^m} \approx \frac{k^4}{6\pi\varepsilon_0^2|E_0^2|}\left|p_j\right|^2 + \frac{k^4\varepsilon_h}{6\pi\varepsilon_0^2 c^2|E_0^2|}\left|m_j\right|^2 + \frac{k^6}{80\pi\varepsilon_0^2|E_0^2|}\left|Q_{jk}^e\right|^2 + \frac{k^6{\varepsilon_h}^2}{80\pi\varepsilon_0^2 c^2|E_0^2|}\left|Q_{jk}^m\right|^2 + \frac{k^8{\varepsilon_h}^2}{1890\pi\varepsilon_0^2|E_0^2|}\left|O_{jkl}^e\right|^2 + \frac{k^8{\varepsilon_h}^3}{1890\pi\varepsilon_0^2 c^2|E_0^2|}\left|O_{jkl}^m\right|^2,$$

(Eq. S1)

where $|E_0|$ is the electric field amplitude of the incident plane wave, k is the wavenumber, and c is the speed of light, ε_h is the permittivity of the host media (air in our case), ε_0 is the permittivity of vacuum, p_j and m_j are the electric (ED) and magnetic dipole moments (MD), (Q_{jk}^e) and (Q_{jk}^m) are the electric and magnetic quadrupoles (EQ and MQ), (O_{jkl}^e) and (O_{jkl}^m) electric and magnetic octupoles (EO and MO).

Supplementary References:

[1] D. Filonov, S. Kolen, A. Shmidt, Y. Shacham-Diamand, A. Boag, and P. Ginzburg, *Volumetric 3D-Printed Antennas, Manufactured via Selective Polymer Metallization*, Physica Status Solidi (RRL) - Rapid Research Letters (2019).

[2] V. D. Burtsev, T. S. Vosheva, A. A. Khudykin, P. Ginzburg, and D. S. Filonov, *Simple Low-Cost 3D Metal Printing via Plastic Skeleton Burning*, Scientific Reports 2022 12:1 **12**, 1 (2022).

[3] R. E. Noskov et al., *Golden Vaterite as a Mesoscopic Metamaterial for Biophotonic Applications*, Advanced Materials **33**, 2008484 (2021).

[4] A. Mikhailovskaya, K. Grotov, D. Vovchuk, A. Machnev, D. Dobrykh, R. E. Noskov, K. Ladutenko, P. Belov, and P. Ginzburg, *Superradiant Scattering Limit for Arrays of Subwavelength Scatterers*, Phys Rev Appl **18**, 054063 (2022).

[5] R. Alaee, C. Rockstuhl, and I. Fernandez-Corbaton, *An Electromagnetic Multipole Expansion beyond the Long-Wavelength Approximation*, Opt Commun **407**, 17 (2018).

[6] A. B. Evlyukhin, C. Reinhardt, E. Evlyukhin, and B. N. Chichkov, *Multipole Analysis of Light Scattering by Arbitrary-Shaped Nanoparticles on a Plane Surface*, Journal of the Optical Society of America B **30**, 2589 (2013).
